# Distinct roles of PilI and ChpC in *Pseudomonas aeruginosa* Chp chemosensory array architecture

**DOI:** 10.1128/aem.01286-26

**Published:** 2026-08-27

**Authors:** Alyssa Kline, Zachary T. Hying, Mackinnley Rybolt, Sonia L. Bardy

**Affiliations:** 1 Department of Biological Sciences, University of Wisconsin Milwaukee 118734, Milwaukee, Wisconsin, USA; Michigan State University, East Lansing, Michigan, USA

**Keywords:** chemotaxis, signal transduction, *Pseudomonas aeruginosa*, cAMP, adaptor, array

## Abstract

Bacteria integrate environmental signals through chemosensory systems to coordinate chemotaxis and make lifestyle decisions, such as surface attachment and biofilm formation. The core sensory complex consists of three essential components: chemoreceptors, histidine kinases, and adaptor proteins. Together, these proteins form extended arrays that result in efficient signal transduction. The Chp chemosensory system of *Pseudomonas aeruginosa* regulates two outputs: direct actuation of type IV pili resulting in twitching motility and modulation of cyclic-AMP production, which regulates pilus biogenesis and acute virulence. A fully functional Chp chemosensory system requires two adaptor proteins, PilI and ChpC. In this study, we investigated the distinct contributions of these adaptors to chemosensory array organization. Using bacterial two-hybrid and fluorescence microscopy, we show that PilI interacts with the histidine kinase and is essential for polar signaling array formation. In contrast, ChpC fails to show direct interaction with the histidine kinase, and the loss of ChpC reduces the number of detectable Chp signaling arrays. Bioinformatic and AlphaFold modeling support the concept of distinct interactions within the array. We integrated these results with previously published data on the roles of the adaptors in signal transduction to propose a model for extended array architecture in which PilI acts as the primary adaptor, while incorporation of ChpC appears to enhance array organization and signaling efficiency.

**IMPORTANCE:**

Bacteria rely on chemosensory systems to detect environmental cues and regulate behaviors such as surface attachment and motility. While many of these systems use a single adaptor protein, others encode multiple adaptors whose roles in array organization and signaling are not well understood. In this study, we show that the two adaptor proteins of the *Pseudomonas aeruginosa* Chp system are distinct, with PilI required for assembly of signaling arrays, and ChpC appears to extend the array and its performance. These findings provide a framework for understanding how multi-adaptor chemosensory systems are structured and how array architecture influences signal integration. More broadly, this work highlights how variations in core signaling components can shape bacterial responses to environmental conditions.

## INTRODUCTION

Chemosensory systems are two-component-like systems that detect extracellular cues and generate coordinated cellular responses through a sensory complex and response regulator. The core sensory complex consists of three types of proteins: (i) chemoreceptors (usually transmembrane proteins) that form trimers of dimers, (ii) histidine kinase (CheA) dimers, and (iii) adaptor (CheW) monomers, which scaffold interactions between chemoreceptors and the histidine kinase, coupling CheA to chemoreceptor control (1–3). Interaction with a ligand induces conformational changes to the cytoplasmic domains of the chemoreceptors. These conformational changes are transduced by the adaptor proteins to the histidine kinase (CheA). Phosphorylated CheA transfers the phosphoryl group to the response regulator, thereby generating the cellular response.

Chemosensory systems exhibit high sensitivity, cooperativity, and the ability to amplify signals, which results from higher-order interactions between multiple core sensory complexes that assemble and function together in large, ordered arrays that have hexagonal and orthogonal symmetry (2, 4). This organizational structure is conserved among membrane-bound and cytosolic chemosensory arrays in bacteria and archaea (1, 5). In *Escherichia coli,* the baseplate of the larger array consists of six-membered rings of either alternating CheW and CheA or six monomers of CheW (6–8). Dimers of CheA have previously been described as “structural staples” that link one hexagonal ring to another, providing structural stability to the array (8). The CheA P5 domain (CheW-like) also interacts with the cytoplasmic tip of chemoreceptors. However, this interaction is not required to induce a conformational change in CheA (2).

The baseplate’s stoichiometry varies among species and plays a key role in fine-tuning chemosensory signaling (8–10). Some species also incorporate additional baseplate-associated proteins, all of which contain a domain that is structurally homologous to CheW (8). These include ParP (a partitioning protein with a CheW-like domain) and CheV (a receiver domain fused to a CheW-like domain) (3, 8, 10–12). Formation and localization of chemosensory arrays ultimately depend on interactions between the proteins that make up the array baseplate and the chemoreceptors.

Unlike the core sensory complex typically found in *E. coli* and other Fla (flagellar motility) class chemosensory systems, the conserved gene organization of F6, F10, type IV pili (Tfp) and alternative (nonmotility) cellular function (ACF) systems indicates the use of multiple adaptor proteins (13). *Pseudomonas aeruginosa* has four complete chemosensory systems, including the Chp system, which is a Tfp-class system with two adaptor proteins, PilI and ChpC. The Chp system contributes to surface sensing and may respond to diffusible ligands, including interspecies ligands (14–16).

At a mechanistic level, the Chp system controls twitching motility and cAMP levels (13, 17–21). These outputs are inextricably linked, as cAMP levels directly modulate the expression of type IV pilus structures (T4P) (21). Input signals are received through the chemoreceptor (PilJ) (16, 22, 23). Upon signal reception, a conformational change is induced at the cytoplasmic tip of PilJ. This conformational change is transduced from PilJ to the histidine kinase (ChpA) (24). Phosphates are then transferred through ChpA to the receiver domains of the CheY-like response regulators, PilG and PilH (25, 26). When phosphorylated, PilG and PilH function antagonistically to modulate twitching motility by acting on the TFP-associated extension (PilB) and retraction (PilU/T) ATPases, respectively (26–28). PilG ~*P* also modulates the adenylate cyclase, CyaB, producing cAMP (21). Twitching direction is determined by asymmetric polar localization of PilG, which corresponds with a positive feedback loop of Chp signaling at the leading pole. Reversals are initiated through the interruption of this feedback loop by PilH ~ P interfering with PilG phosphorylation (27).

The interaction between chemoreceptors and the histidine kinase is essential for signal transduction in chemosensory systems. As discussed above, in many chemosensory systems, this interaction is mediated by a single adaptor protein, CheW (3, 28). The Chp chemosensory system, like other Tfp-class chemosensory systems in *Gammaproteobacteria*, differs from this paradigm and encodes two CheW-like adaptor proteins: PilI and ChpC (18). Previous studies in *P. aeruginosa* PAK demonstrated that PilI and ChpC are not equivalent. Loss of PilI results in a complete eradication of cAMP generation through CyaB, and cells that are null for twitching motility. In contrast, loss of ChpC results in a partial phenotype (18, 21).

The roles of these adaptor proteins in Chp array architecture are not fully understood and may impact signal transduction. Understanding the roles of PilI and ChpC has important implications for understanding chemosensory systems with multiple adaptor proteins as well as understanding *P. aeruginosa* regulation and virulence. We sought to define the roles of PilI and ChpC in organizing Chp chemosensory arrays and advance a model for Chp array formation and implications for downstream signaling.

## MATERIALS AND METHODS

### Bacterial strains, plasmids, and growth conditions

*P. aeruginosa* PAO1 (Iglewski strain) and *E. coli* strains were grown in Lysogeny Broth (LB) at 37°C with aeration in liquid or on 1.5% agar plates unless otherwise indicated. Strains, plasmids, and primers used in this study are listed in Tables S1, S2, and S3, respectively. Antibiotic concentrations for *P. aeruginosa* were gentamicin 50 µg mL^−1^ and tetracycline 75 µg mL^−1^. Antibiotic concentrations for *E. coli* were ampicillin 100 µg mL^−1^, kanamycin 50 µg mL^−1^, gentamicin 10 µg mL^−1^, and tetracycline 10 µg mL^−1^.

### Construction of PAO1 deletion and expression strains

In-frame deletions of *pilA, cyaB, pilI,* and *chpC* were generated using overlap extension PCR. PCR products of the resulting deletion alleles were ligated into pEX18Tc and transformed into *E. coli* S17-1 by electroporation. Plasmids were transferred to *P. aeruginosa* through biparental mating (22), and merodiploids were selected on LB with tetracycline (75 µg mL^−1^) and chloramphenicol (5 µg mL^−1^) to counter-select against the donor strain. Resolution of merodiploids was achieved through 10% sucrose counter-selection. Second recombinants were screened for deletion by PCR.

Expression plasmids were made by PCR amplification of *pilI*, *chpC,* and *cyaB_R412H_*. These PCR products were ligated into pSB109 (*pilI* and *chpC*) or pJN105 (*cyaB_R412H_*). Constructs were confirmed by sequencing, and purified plasmids were electroporated into *P. aeruginosa* strains.

### Analysis of Tfp-class chemosensory systems distribution in *Gammaproteobacteria*

Members of *Gammaproteobacteria* encoding Tfp chemosensory proteins were identified from a survey of the MiST database (29), and the genomic neighborhood was searched for CheW-like proteins (Pfam: PF01584). Species used in this analysis were selected on the basis of their inclusion in the previously published phylogenetic tree (30).

### Sequence retrieval and multiple sequence alignment

Protein sequences for *P. aeruginosa* PilI and ChpC were obtained from the Pseudomonas Database (31). CheW-like proteins containing a putative CheA interaction surface from *E. coli, Thermotoga maritima, Synechocystis sp., Myxococcus xanthus, Rhodospirillum rubrum, Borrelia burgdorferi,* and *Treponema pallidum* were identified from the Conserved Domain Database (32), and the amino acid sequences for these proteins were downloaded in FASTA format from NCBI. Species were selected to represent the most diverse members. These sequences were aligned with PilI and ChpC using CLUSTAL Omega (https://www.ebi.ac.uk/jdispatcher/msa/clustalo) through EMBL-EBI’s web server (33).

### Construction of bacterial two-hybrid expression vectors

PCR-amplified *pilI*, *pilJ*, truncated *chpA* (*chpA_cheA_*, encoding amino acids 1668–2349)*,* and *chpC* were digested with appropriate restriction endonucleases and ligated into pKT25, pKNT25, pUT18, or pUT18C (bacterial adenylate cyclase two-hybrid [BACTH] vectors). Plasmids with sequenced inserts were electroporated into DHM1. Transformed cells were selected on LB ampicillin (100 µg mL^−1^) and/or LB kanamycin (50 µg mL^−1^). Experimental and control DHM1 strains are listed in Table S1.

### Protein–protein interaction assays

Protein interactions were determined using the bacterial adenylate cyclase two-hybrid system (Euromedex). Components of the Chp signaling array were fused to either the T18 or T25 fragment of *Bordetella pertussis* adenylate cyclase as described in the previous section. Protein interaction results in reconstitution of the adenylate cyclase, as evidenced by increased Miller Units (β-galactosidase assays).

DHM1 strains were grown overnight in LB with ampicillin (100 µg mL^−1^) and kanamycin (50 µg mL^−1^) at 30°C with aeration. For β-galactosidase assays, overnight cultures were diluted and grown to mid-log phase (OD_600_ 0.4–0.9) at 30°C in LB with ampicillin, kanamycin, and 0.5 mM IPTG to induce expression of fusion proteins. β-galactosidase assays were performed as previously described (34). For each strain, the assay was performed on three biological samples on three separate days, with each sample assayed with three technical replicates.

### Microscopy and data analysis

Individual colonies of 24-hour surface-grown cells expressing PilJ-mCherry were resuspended in 100 μL of PBS in black microcentrifuge tubes and incubated on ice for 15 minutes. To induce adherence of bacterial cells to the slide, 4 μL of the resuspension was directly added to a poly-lysine coverslip. The cells were washed with PBS, 4 μL of PBS was added to the slide, and the coverslip was placed over the liquid. Cells were then visualized under a Nikon Eclipse 90i upright microscope, and images were captured using a Hamamatsu Orca-Flash 4.0 digital camera with a 300 ms exposure; LUTs were set to 600 (G = 1.0) in NIS-Elements software version 4.13. Fluorescence profiles were generated using ImageJ (version 1.53k; https://imagej.nih.gov/ij/), and demographs were constructed in R (version 4.1.3; R Foundation for Statistical Computing; http://www.r-project.org) as described (35).

### *P. aeruginosa* phenotypic assays

#### Twitching motility assay

Isolated *P. aeruginosa* colonies were stab inoculated into LB plates (1% agar). Plates were incubated at 37°C for 40 hours as previously described (22). After agar removal, the diameter of the twitching zones was measured. Nine colonies were assayed per strain.

#### Relative intracellular cAMP levels

β-Galactosidase assays to determine the relative levels of intracellular cAMP were performed using surface-grown *P. aeruginosa* strains containing the *lacP1-lacZ* reporter construct integrated into their genome (21, 22). After overnight incubation at 37°C, cells were scraped from LB agar (1.5%) and resuspended in LB broth. OD_600_ was measured, and cultures were diluted to an OD_600_ 0.4–0.8. β-galactosidase assays were performed as previously described (21). Each strain was assayed three times, with three technical replicates for each data point.

#### PilA expression levels

To determine whole-cell levels of PilA, strains were streaked for lawn growth and incubated overnight at 37°C. Cells scraped from the plate were resuspended in 0.15 M NaCl + 0.2% formaldehyde and normalized to 0.6 (OD_600_). An equal amount of 2× SDS-PAGE loading dye was added to the cell suspension, and whole-cell lysates were incubated at 95°C for 10 minutes. Protein samples were separated by 15% SDS-PAGE and transferred to a polyvinylidene difluoride (PVDF) membrane. A western blot was performed by probing membranes with anti-PilA (1:10,000) followed by goat anti-rabbit conjugated to peroxidase (1:100,000). Three biological replicates were prepared for each strain and independently quantified using ImageJ software, where each replicate originated from a distinct colony.

#### Surface piliation levels

Surface-grown cells were scraped from the plate and resuspended in 0.15 M NaCl + 0.2% formaldehyde. Cell suspensions were normalized to 20 (OD_600_). Surface proteins, including PilA, were sheared from the bacterial surface by vortexing for 30 minutes. Bacterial cells were removed by centrifugation at 12,000 × *g* for 5 minutes. Sheared proteins were precipitated in 100 mM MgCl_2_ overnight at 4°C and pelleted by centrifugation at 16,873 × *g* for 25 minutes (21). Sheared proteins were resuspended in 50 µL PBS and SDS-PAGE loading buffer and resolved on 15% SDS-PAGE. After staining with Coomassie Brilliant Blue (G-250), 4% perchloric acid gels were photographed on a Fotodyne Luminary System. Three biological replicates were prepared and quantified using ImageJ software.

### Statistical analysis

Data were analyzed using a pairwise *t*-test with multiple variances, and one or two tails as appropriate. The Holm–Bonferroni method was used to correct for multiple pairwise comparisons.

## RESULTS

**Fig 1:**
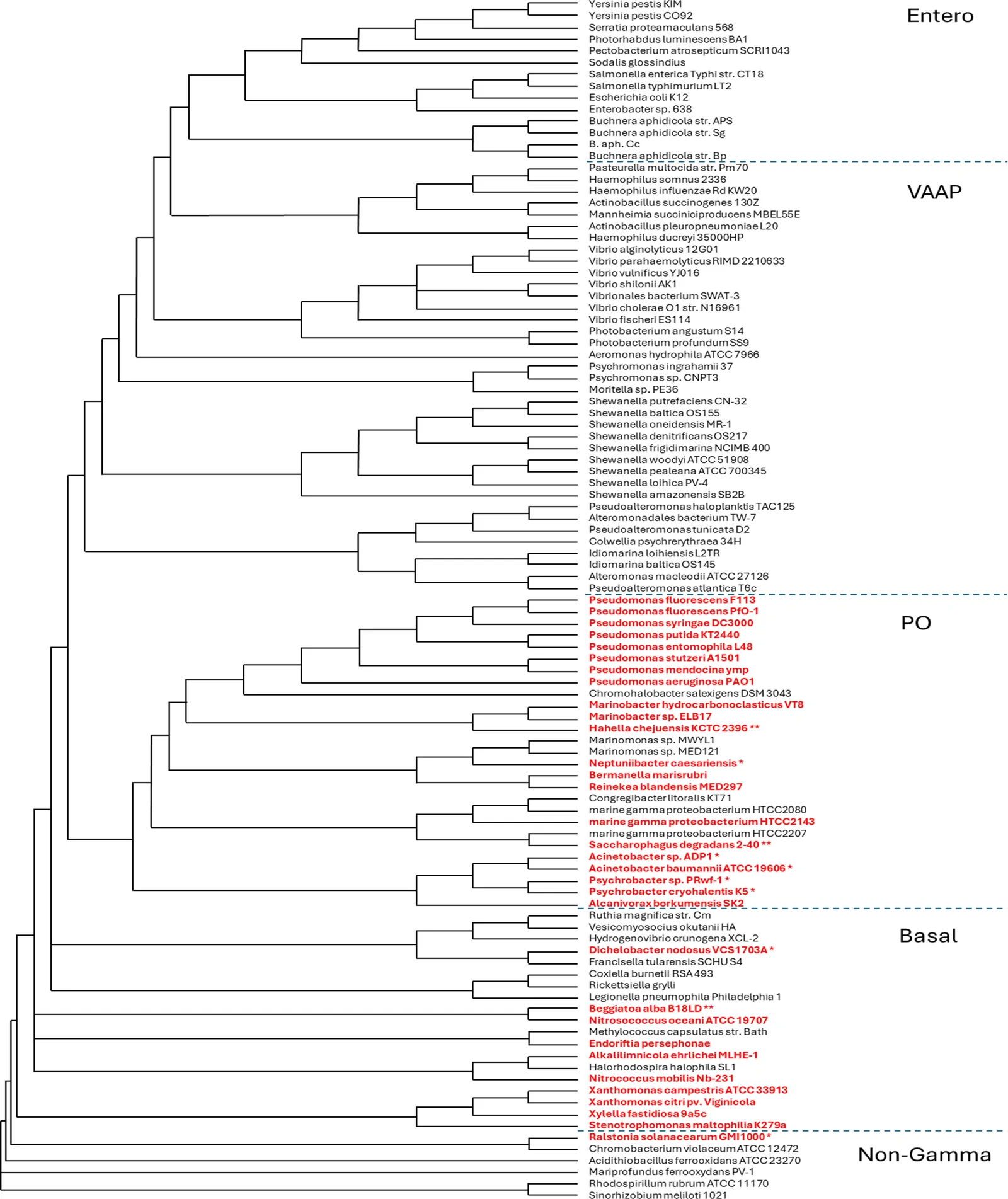
Phyletic distribution of Tfp systems in Gammaproteobacteria. Cladogram depicting the evolutionary relationships of Gammaproteobacteria as defined in https://pmc.ncbi.nlm.nih.gov/articles/PMC2863478/. The length of branches does not represent evolutionary distance. “Entero” = *Enterobacterales*, “VAAP” = *Vibrionales*, *Alteromonadales*, *Aeromonadales*, and *Pasteurellales*, “PO” =*Pseudomonadales* and *Oceanospirillales*, and “Basal” = paraphyletic group of the most anciently diverging lineages in the class. Species highlighted in red were found to contain Tfp chemosensory genes. Systems encoding one CheW-like adaptor are indicated by a single asterisk. Two asterisks indicate the presence of multiple Tfp-class chemosensory operons.

The members of the phylogenetic tree of *Gammaproteobacteria* from Williams et al. (30) were cross-referenced with the MiST database (29) to identify species with Tfp-class chemosensory systems and the number of CheW-like adaptors encoded within them. Tfp-class systems were found to be clustered within two distinct clades ("PO” and “Basal”), and of those systems identified, approximately 77% were found to encode two adaptors (Fig. 1 and Table S4). The previous phylogenetic study also included two species each from the *Betaproteobacteria* and the *Alphaproteobacteria* (30). Of these four species, the betaproteobacterium *Ralstonia solanacearum* was the only one with a Tfp-class chemosensory system, which encodes a single adaptor (30). Further investigation of Tfp-class chemosensory systems revealed that these systems are found among all orders in *Betaproteobacteria,* and they almost exclusively contain one adaptor, unlike those in the *Gammaproteobacteria* (Table S3). Previous bioinformatics studies have only identified Tfp-class systems in *Gammaproteobacteria* and *Cyanobacteria* (29).

**Fig 2:**
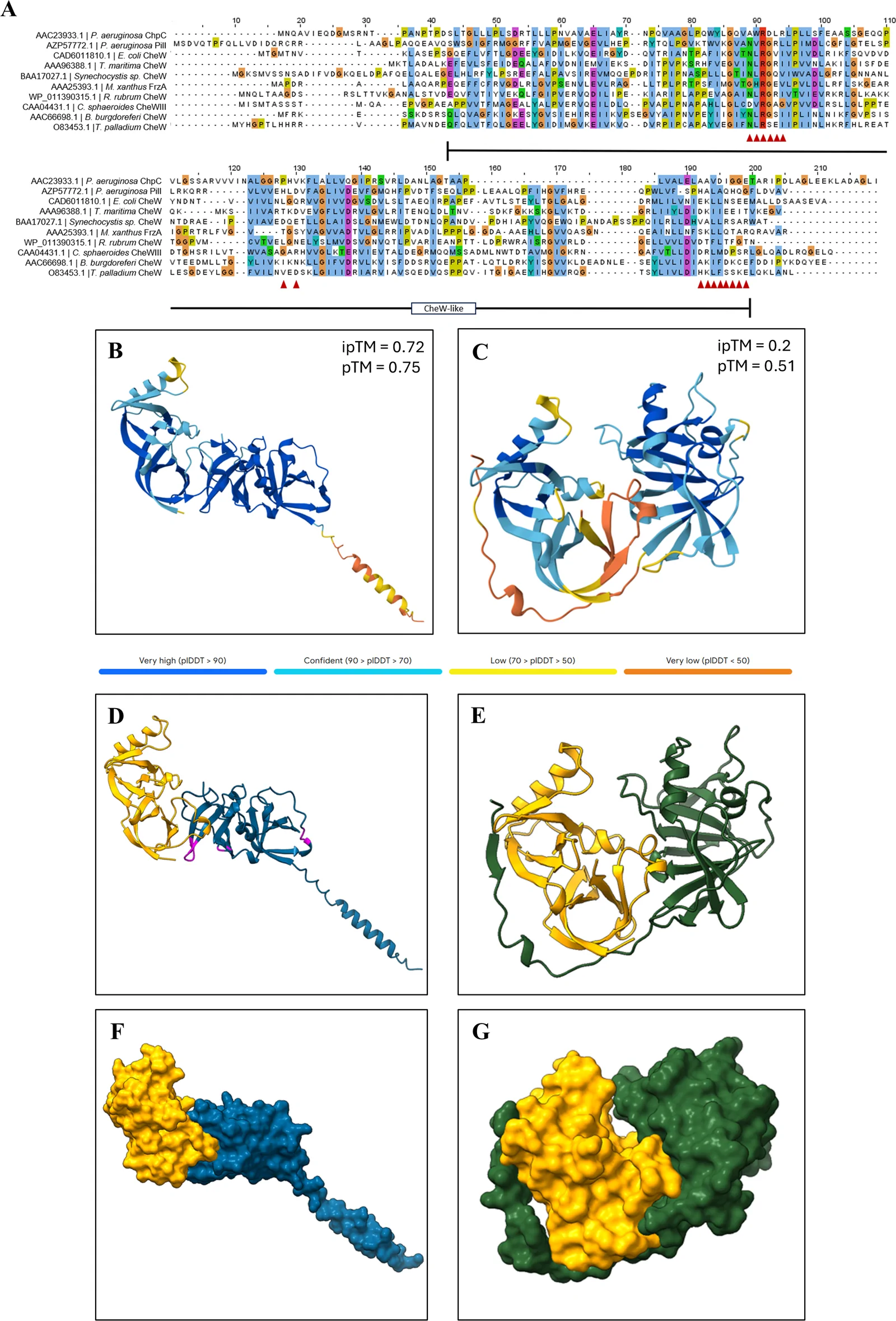
Predicted complexes of adaptors with the P5 domain of ChpA. (**A**) Multiple sequence alignment of select CheW proteins (NCBI-CDD: cd00732) containing a putative CheA interaction surface with *P. aeruginosa* PilI and ChpC. Residues associated with the CheA interaction surface are indicated by the red triangles at the bottom of the alignment. Residues colored according to the ClustalX color scheme: hydrophobic (blue), positive charge (red), negative charge (magenta), polar (green), cysteine (pink), glycine (orange), proline (yellow), aromatic (cyan), unconserved (white). (**B** and **C**) Ribbon diagrams of AlphaFold-predicted complexes of the ChpA—P5 domain and (B) PilI or (C) ChpC. Shown are the top-ranked ribbon diagrams of the five models proposed by AlphaFold and colored based on predicted local confidence score (pLDDT; color legend is provided below panels B and C). Confidence for the overall structure of the complex is represented by the pTM score (>0.5 indicates high confidence). Accuracy of the predicted relative positions of the subunits forming the protein–protein complex is represented by the ipTM score (>0.8 represents high-quality predictions, <0.6 suggests a likely failed prediction). Ribbon diagrams (**D** and **E**) and surface diagrams (**F** and **G**) of the AlphaFold models with individual proteins highlighted for clarity: P5 domain of ChpA (yellow), PilI (blue), ChpC (green), and putative CheA interaction surface (magenta).

In *P. aeruginosa*, the Chp core signaling array is proposed to comprise a single chemoreceptor (PilJ), a single histidine kinase (ChpA), and two adaptors, PilI and ChpC. A pairwise alignment of ChpC and PilI protein sequences reveals 20.28% identity, while pairwise alignments of PilI and ChpC with *E. coli* CheW have 21.57% and 22.08% identity, respectively (36). Interestingly, according to the NCBI conserved domain database (32), PilI contains conserved residues indicating a putative CheA interaction surface, while ChpC does not (Fig. 2A). This distinction prompted us to further examine the predicted structures of PilI and ChpC and their potential for interactions with ChpA. Protein sequences for *P. aeruginosa* PilI, ChpC, and the P5 domain of ChpA were run through AlphaFold (https://alphafoldserver.com/) (37) to generate predicted protein complexes. The top-ranked AlphaFold model of PilI interaction with the P5 domain of ChpA is shown in Fig. 2B, D, and F, while Fig. 2C, E, and G show AlphaFold models between ChpC and the P5 domain of ChpA. The AlphaFold analysis of the overall accuracy of these complexes (pTM score) suggests high confidence for both PilI-ChpA and ChpC-ChpA models, although it is worth noting the pTM score for ChpC-ChpA is barely above the 0.5 cutoff. In this instance, the ipTM scores, which indicate accuracy of the predicted relative positions of the subunits, are more informative. The ChpA-ChpA model has a failing score (ipTM = 0.2) and introduces uncertainty in the predicted structure of ChpA, as seen in the pLDDT score. In contrast, the predicted PilI-ChpA interaction has an ambiguous ipTM score, as scores between 0.6 and 0.8 indicate the model may or may not be correct. The predicted local confidence in the PilI-ChpA interface residues is high (based on pLDDT scores), suggesting these regions of the model are robust. Overall, while the computational predictions are not definitive regarding PilI and ChpA interactions, they raise significant questions regarding the interactions between ChpC and the P5 domain of ChpA. We therefore sought to determine the role of the dual adaptors in array formation by investigating protein interactions of array components and protein localization patterns in key deletion strains.

### Interactions between core signaling array components

**Fig 3:**
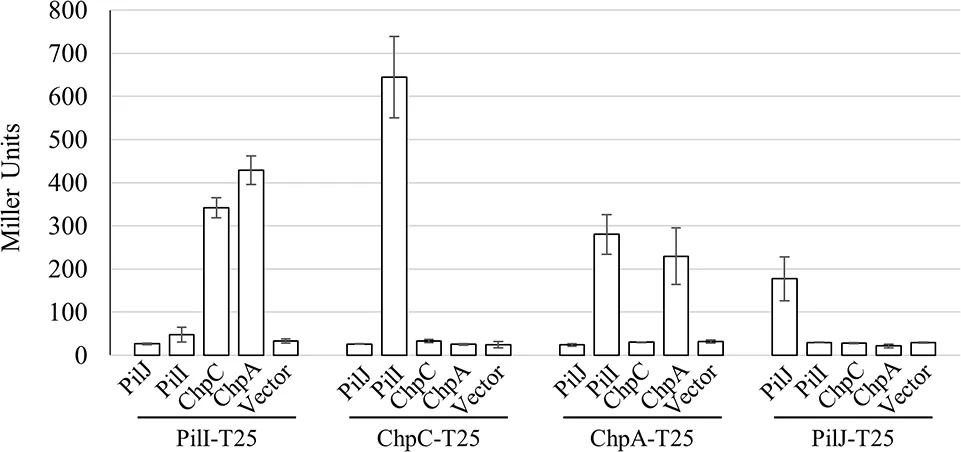
Interactions between components of the Chp core signaling array. PilI, ChpC, ChpA_CheA_, and PilJ were cloned into pKNT25. The other predicted core signaling components were cloned into pUT18. “Vector” indicated empty pUT18. Plasmids were co-transformed into strain DHM1, and interactions were quantified using β-galactosidase assay. The data result from three samples, and each sample is an average of three technical replicates. Error bars indicate the standard error of the mean.

The BACTH system was used to determine the interaction partners of each adaptor protein of the Chp system. The BACTH system works by reconstituting the active site of the adenylate cyclase CyaA from *Bordetella pertussis* (34, 38). Genes encoding adaptors were cloned into each of the vectors encoding either the T25 or T18 fragment. Our data showed that PilI and ChpC interact with each other but do not show self-interaction (Fig. 3). All PilI-PilI and ChpC-ChpC combinations resulted in low β-galactosidase activity, like the empty vector negative controls.

We also tested the interactions of the adaptors with other core chemosensory components. Truncated *chpA* (*chpA_cheA_*) and *pilJ* were cloned into each of the four BACTH vectors and transformed into experimental DHM1 strains with plasmids expressing PilI or ChpC. The histidine kinase ChpA is a large and complex protein that contains eight predicted phosphotransfer domains, an ATPase domain, a dimerization domain, and a receiver domain (26). Previous studies in *Pseudomonas* have shown poor expression of the full-length protein (18). Because of this, we cloned the portion of the gene containing components necessary for chemosensory function based on homology to the *E. coli* histidine kinase CheA into each BACTH vector for our interaction studies: ChpA_CheA_ (amino acids 1,668–2,349). This truncation was used in a previous study that determined the activity of the phosphotransfer sites (26). This truncation includes one phosphotransfer domain, an ATPase domain, a P5 domain, and the dimerization domain corresponding to the globular CheA core. In our interaction studies, ChpA_CheA_ interacted with PilI but not ChpC, and this result is in alignment with *in silico* data from NCBI’s Conserved Domain Database (NCBI-CDD [32]). We examined PilI and ChpC protein sequences through this database and found that PilI had a putative CheA interaction surface (cd00732) while ChpC did not. A recent study categorized CheW-like domains into six distinct classes (39). PilI most closely aligns with CheW.IB (Class 1), while ChpC aligns more with CheW.IA (Class 6). Interestingly, Class 6 domains exhibited strong phyletic coupling to chemoreceptors, but not to any CheA proteins, while Class 1 domains had strong associations with both. This prior analysis aligns well with our experimental data and supports our current model, although we have yet to fully explore potential interactions between ChpC and untested regions of ChpA.

We were unable to detect interactions between the adaptor proteins and PilJ. To ascertain whether the T18 and T25 fusions on PilJ were interfering with the predicted interactions between the adaptors and the chemoreceptor, we assayed PilJ–PilJ interactions. PilJ showed strong self-interactions, likely due to the formation of homodimers as reported for other chemoreceptors (40). We expect that the failure to demonstrate an interaction between PilJ and the baseplate components results from spatial constraints. Both amino-terminal and carboxy-terminal fusions of T25 or T18 on PilJ localize near the inner membrane. This is sufficient to bring the fragments into close proximity for testing PilJ/PilJ and PilJ/PilA interactions (26, 41). In contrast, the trimer association domain of PilJ, where ChpC and PilI interact, extends approximately more than 28 nm into the cytoplasm (42, 43). We expect that this distance was too great to allow the T25 and T18 fragments to come into proximity when PilJ/PilI or PilJ/ChpC interactions were tested. Similar results have previously been reported in *Helicobacter pylori* (3), where the BACTH system was unable to detect interaction between TlpB and CheW. Both PilJ and TlpB are transmembrane chemoreceptors in the 40H MCP class (44).

### PilJ polar localization is dependent on PilI, but not ChpC

**Fig 4:**
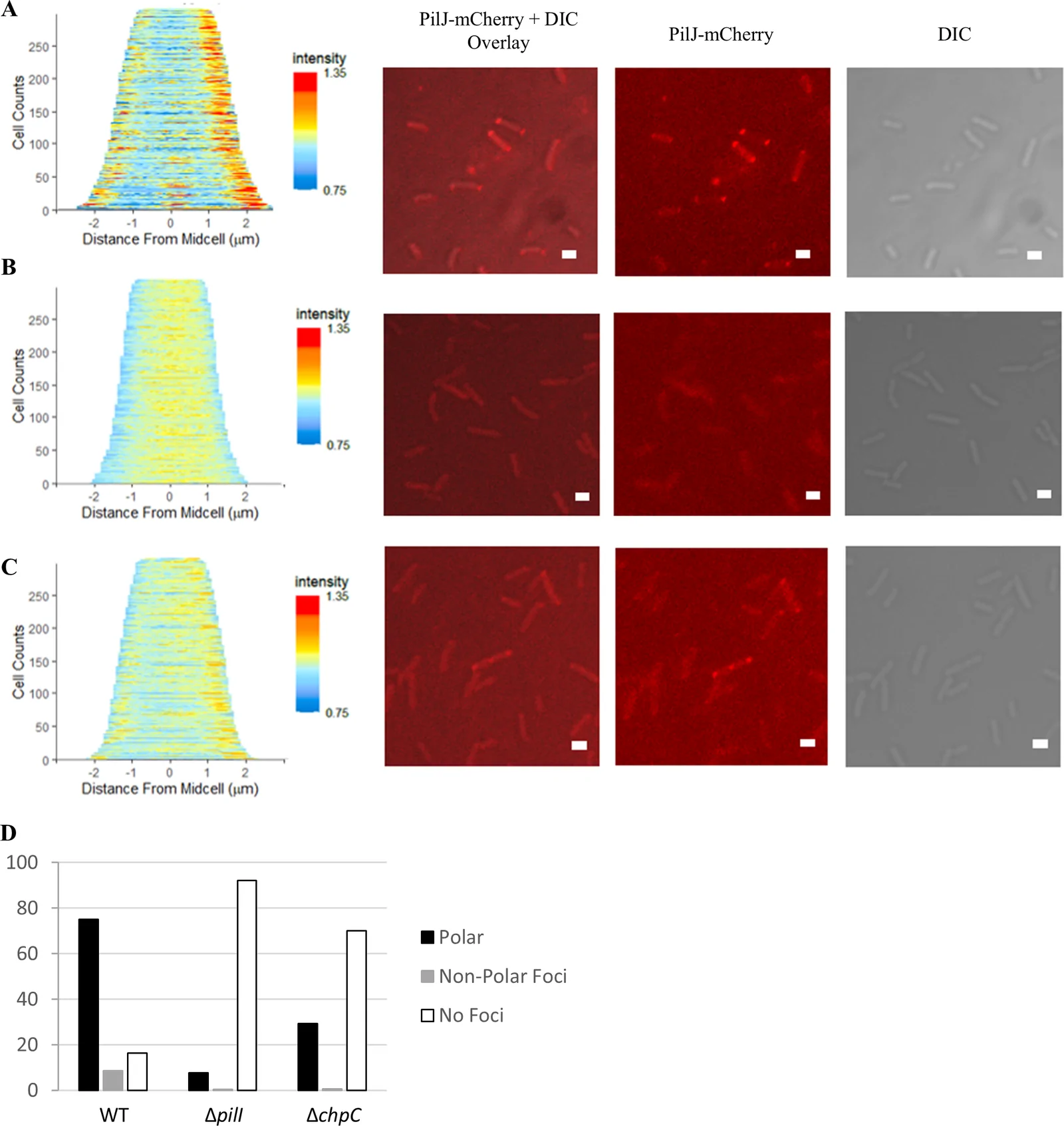
Localization of PilJ-mCherry in *P. aeruginosa* cells. (**A**) PAO1 PilJ-mCherry, (**B**) *ΔpilI* PilJ-mCherry, and (**C**) *ΔchpC* PilJ-mCherry. For each strain, 300 cells are represented as lines and ordered according to their length in demographs. The mCherry fluorescence is indicated by colored pixels at the corresponding cell position relative to midcell, as indicated on the Y-axis. The scale to the right of the demographs indicates fluorescence intensity and corresponding colors. On the right side of each panel is a fluorescent micrograph of the indicated strains expressing PilJ-mCherry fusions. The scale bar indicates 1 μm. (**D**) Quantitation of PilJ-mCherry foci in the indicated strains.

To further characterize the relationship between the adaptor proteins and the chemoreceptor, we localized PilJ using fluorescent microscopy. PilJ is polar localized and required for twitching motility and regulation of intracellular cAMP (21, 45). We generated a PilJ-mCherry fusion expressed from its native locus on the chromosome (46). In wild-type cells, PilJ-mCherry localized to the poles of the cells in agreement with previous reports (Fig. 4A and D) (45). Deletion of *pilI* resulted in a loss of PilJ-mCherry foci and an increase in background fluorescence throughout the cell length (Fig. 4B and D). In the *chpC* deletion strain, a small percentage of cells retained polar PilJ-mCherry foci when compared to wild type (Fig. 4C and D). These data demonstrate that ChpC is not an essential component in array architecture.

### Functional roles of adaptor proteins

It has previously been shown in *P. aeruginosa* PAK that the Chp adaptor proteins had functional differences impacting both cAMP levels and twitching motility (21). Our data agree with these results and allow us to integrate array architecture with function.

### PilI is required for Chp signal transduction

**Fig 5:**
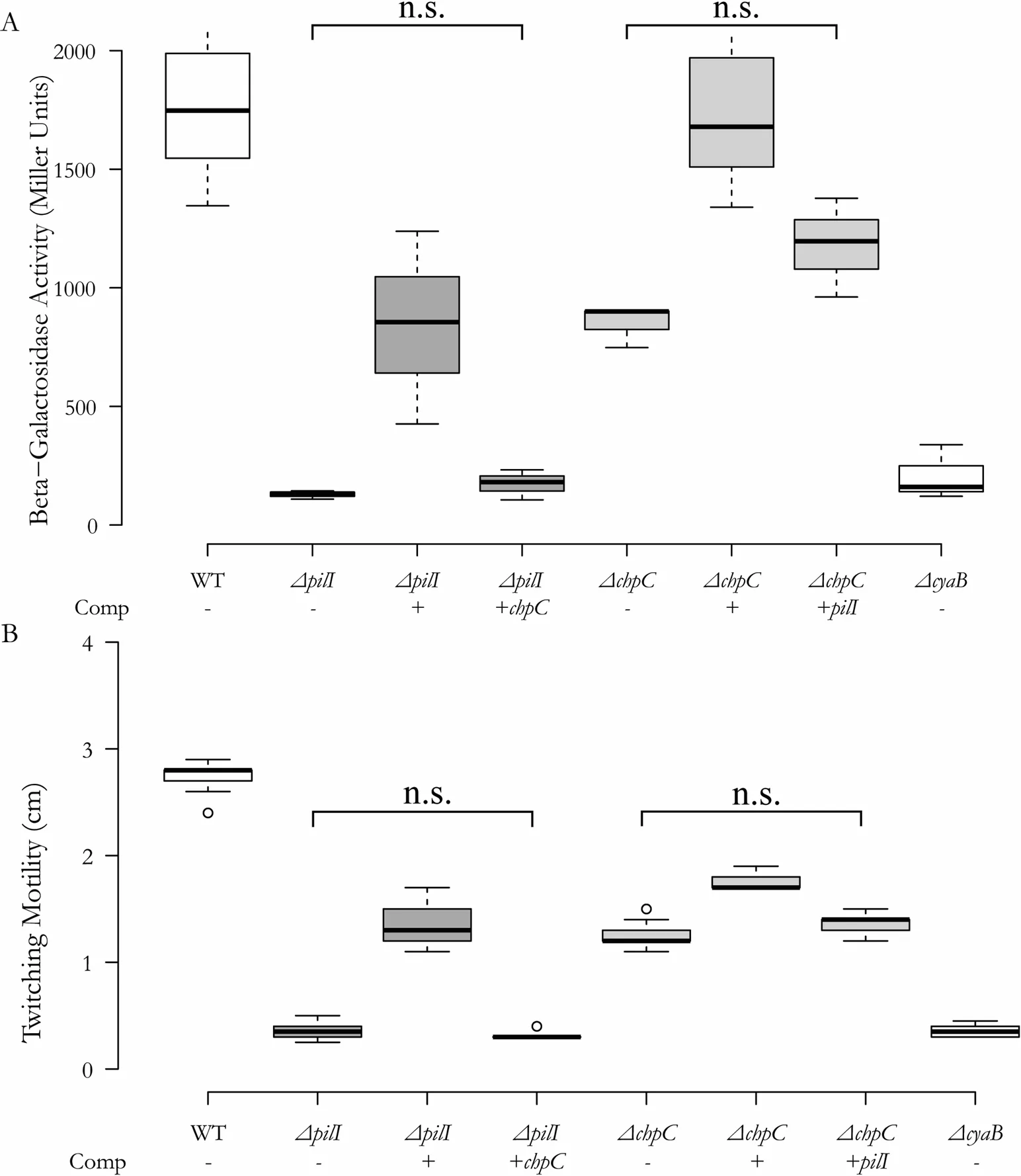
cAMP and twitching motility levels in *P. aeruginosa* strains lacking Chp adaptor proteins. (**A**) Indicated strains were assayed for β-galactosidase activity, an indicator of intracellular cAMP bound to Vfr. Center lines show the medians; box limits indicate the 25th and 75th percentiles as determined by R software; whiskers extend 1.5 times the interquartile range from the 25th and 75th percentiles. *n* = 3 sample points. Each data point is an average of three technical replicates. (**B**) Twitching motility of the indicated strains as determined by the diameter of the twitching motility zones. Center lines show the medians; box limits indicate the 25th and 75th percentiles as determined by R software; whiskers extend 1.5 times the interquartile range from the 25th and 75th percentiles; outliers are represented by dots. *n* = 9 colonies. n.s. indicates an insignificant difference *P* > 0.05.

We assessed relative intracellular levels of cAMP and twitching motility in *ΔpilI* (Fig. 5). There were significantly lowered levels of intracellular cAMP (*P* = 0.013), comparable to the *ΔcyaB*-negative control for cAMP production (*P* = 0.181). Twitching motility was also decreased in *ΔpilI* (*P* = 1.14e-14), similar to the *ΔpilA*-negative control that lacks the primary pilin subunit (*P* = 0.346), and consistent with previous studies (21, 41).

Complementation of *pilI* restored cAMP and twitching motility to ~50% of WT (Fig. 5). This partial complementation is likely due to one of two possibilities. First, the incorrect stoichiometry of the core signaling complex components due to PilI expression from a non-native promoter could lead to inefficient array formation. Second, when expressed from pSB109:*pilI*, PilI contains an N-terminal 6X-His tag, which may interfere with efficient chemosensory array formation. Interestingly, expression of ChpC in *ΔpilI* did not complement PilI (Fig. 5), further demonstrating that ChpC is not functionally equivalent to PilI.

### ChpC is not essential for Chp signal transduction

We also tested *ΔchpC* for cAMP production and twitching motility as evidence of signal transduction. As previously reported (21), *ΔchpC* shows reduced levels of intracellular cAMP (~48% of WT; *P* = 0.031) and reduced twitching motility (~46% of WT; *P* = 1.30e-13) (Fig. 5A and B). Complementation of *chpC* restored cAMP to WT levels and partially restored twitching motility. Partial restoration of twitching motility could be due to similar reasons as presented above for *pilI* complementation; however, this is contradicted by the presence of WT levels of cAMP. Complementation of *ΔchpC* with *pilI* expressed in *trans* showed a slight, but not statistically significant, increase in cAMP levels, suggesting that overexpression of *pilI* could partially restore signaling in the absence of ChpC (Fig. 5).

### Excess cAMP restores twitching motility in *ΔchpC*

**Fig 6:**
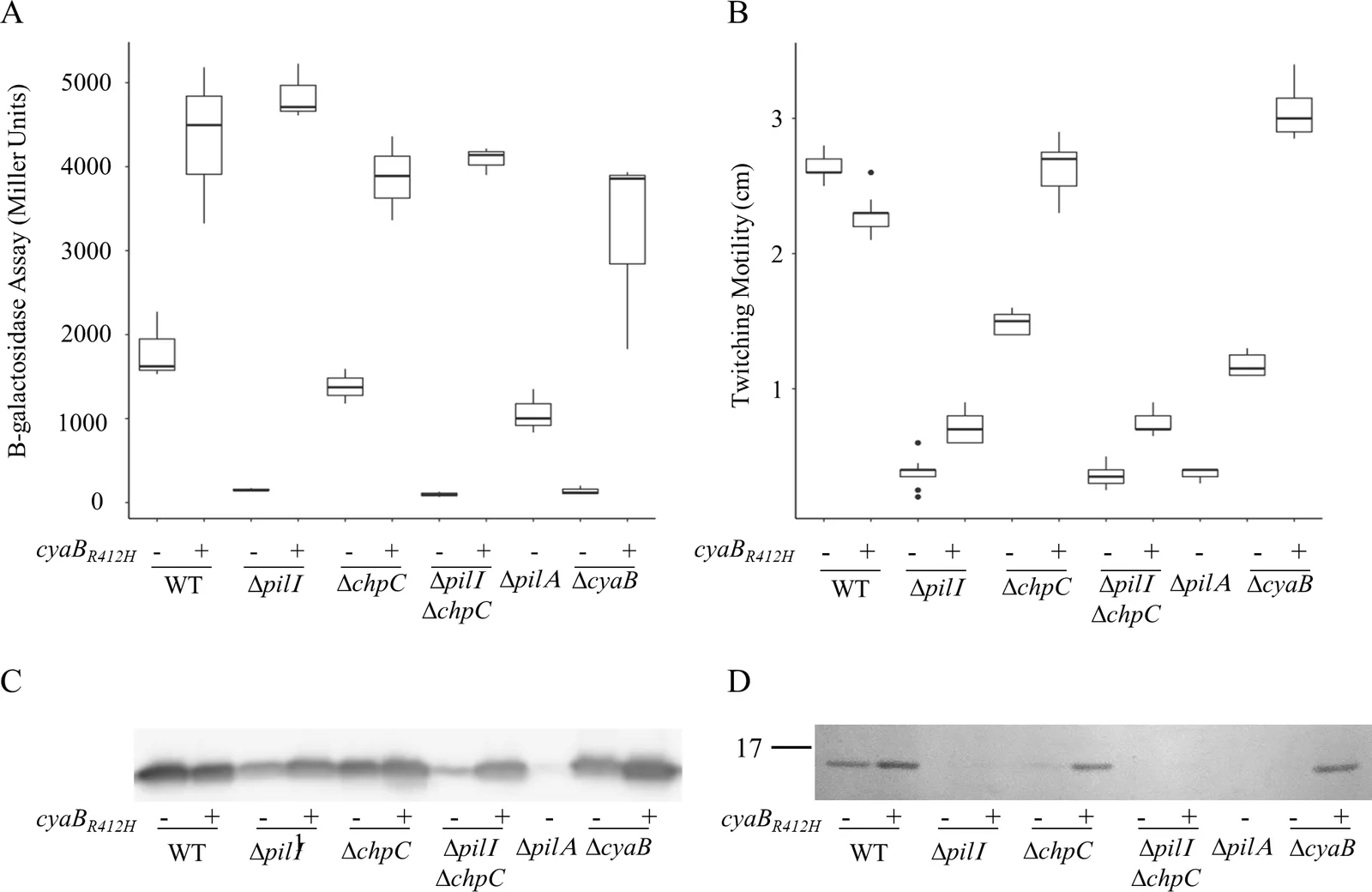
Expression of *cyaB_R412H_* in *P. aeruginosa* strains lacking Chp adaptor proteins. (**A**) Indicated strains were assayed for β-galactosidase activity, an indicator of intracellular cAMP bound to Vfr. *N* = 3 data points. Each data point is an average of three technical replicates. (**B**) Twitching motility of the indicated strains as determined by the diameter of the twitching zones. *N* = 9 individual data points. (**C**) Whole-cell levels of PilA of the indicated strains as determined by western blotting. (**D**) Surface levels of PilA from the indicated strains as determined by SDS-PAGE. In panels A and B, center lines show the medians; box limits indicate the 25th and 75th percentiles as determined by R software; whiskers extend 1.5× the interquartile range from the 25th and 75th percentiles; outliers are represented by dots.

Because the Chp chemosensory system modulates cAMP production, thereby impacting expression of pilus assembly and motor genes, we were unable to determine the effects of adaptor deletions solely on twitching motility. To circumvent this, we expressed a constitutively active point mutant of CyaB (47). CyaB_R412H_ produces cAMP independent of Chp chemosensory signaling and has been used to overcome cAMP deficiencies in previous studies (24). When *cyaB_R412H_* was expressed, it produced levels of cAMP (*P* > 0.05) that were at least twice the level compared to WT cells (Fig. 6A).

This excess level of cAMP in *ΔchpC*(*cyaB_R412H_*) and *ΔcyaB*(*cyaB_R412H_*) resulted in the restoration of twitching motility to WT level (*P* = 0.500) (Fig. 6B), demonstrating that ChpC is not required for signal transduction to support twitching motility when excess cAMP is present.

A novel double deletion strain that lacks both adaptors (*ΔpilIΔchpC*) is null for both cAMP levels and twitching motility, phenocopying the *ΔpilI* deletion strain. In *ΔpilI*(*cyaB_R412H_*) and *ΔpilIΔchpC*(*cyaB_R412H_*), increased intracellular cAMP does not restore twitching motility to WT levels, highlighting the role of PilI in array formation and motility (*P* < 0.0001; Fig. 6B). A slight increase in twitching motility correlates with higher whole-cell PilA (Fig. 6C), as CyaB_R412H_ expression boosts PilA levels in all strains. cAMP restoration is known to enhance T4P biogenesis and motor function gene expression (*pilB* and *pilT/U*) (48), and residual motor activity without Chp signaling has been reported (25). These factors likely explain the modest twitching increase in cAMP-replete cells. While PilA subunits on the surface increased with cAMP in *ΔchpC*(*cyaB_R412H_*) and *ΔcyaB*(*cyaB_R412H_*) (Fig. 6C), *ΔpilI*(*cyaB_R412H_*) and *ΔpilIΔchpC*(*cyaB_R412H_*) showed a smaller increase in surface piliation relative to whole-cell PilA levels (Fig. 6C and D).

## DISCUSSION

**Fig 7:**
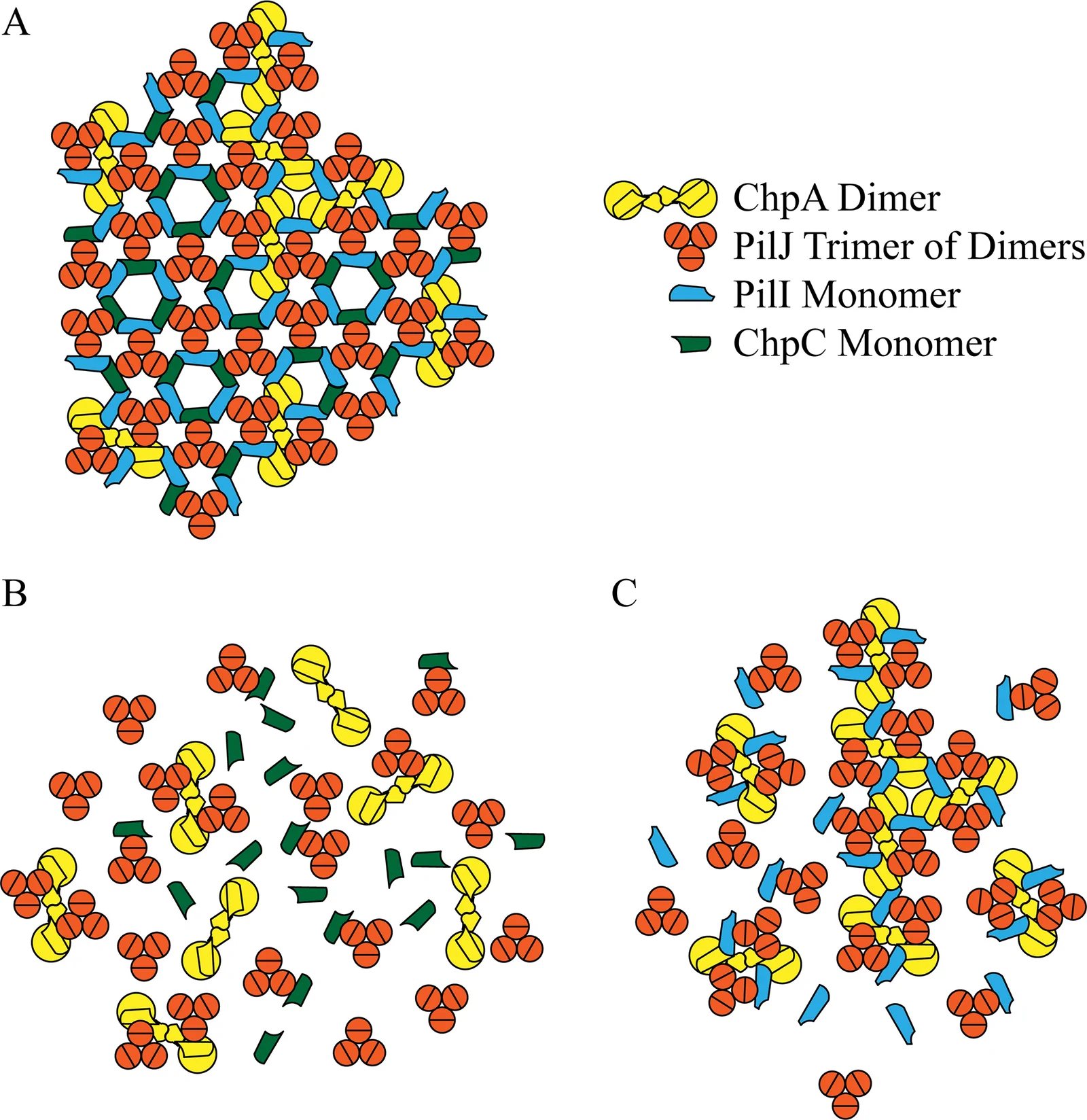
Chp chemosensory array model. ChpA dimers are shown in yellow, PilJ trimers of dimers are shown in orange, PilI is shown in light blue, and ChpC monomers are shown in dark green. (**A**) Modeled Chp chemosensory array in PAO1 WT based on BACTH interaction data. (**B**) Modeled Chp chemosensory array in PAO1 *ΔpilI* strain. (**C**) Modeled Chp chemosensory array in PAO1 *ΔchpC* strain.

The Chp signaling array of *P. aeruginosa* controls two regulatory outputs through the chemoreceptor, PilJ, and histidine kinase, ChpA (18, 21). Here, we present our newfound understanding of the structural roles of two adaptor proteins in array formation and the connection to signal transduction. We used bacterial two-hybrid and fluorescence microscopy data (Fig. 3 and 4) to generate a model of Chp chemosensory arrays that aligns with the physiological roles of the adaptor proteins. Figure 7A models a Chp chemosensory array in WT. As a starting point, we based the model on the P6 hexagonal pattern of the baseplate and regular spacing of chemoreceptors that is conserved among bacteria and archaea (1, 49). We also attempted to model the Chp chemosensory array using the P2 model (50) but could not align our data with this model. To satisfy the P2 array architecture, PilI would need to interact with itself and/or ChpC would interact with the histidine kinase; however, neither of these possibilities is supported by the data herein. Therefore, in line with the P6 model, we expect that each hexagonal ring consists of three PilI monomers interspaced by a combination of ChpA_P5_ and/or ChpC. We predict that a hexagonal ring consisting of a 3:1:2 ratio of PilI:ChpA_P5_:ChpC would be the most effective at signal transduction to ChpA, as it integrates signal from five PilJ receptors into a single ChpA; however, it is likely that hexagonal rings consisting of 3:3:0, 3:2:1, 3:1:2, and 3:0:3 ratios of PilI:ChpA_P5_:ChpC all exist within one array. The interchangeability of each type of hexagonal ring lends to a dynamic, sensitive, structurally flexible, but stable array. Our model includes PilJ organized in trimers of dimers, as previously reported for chemoreceptors (49, 51). While limited structural studies have been done on PilJ, this chemoreceptor has a high degree of homology with Tsr in the cytoplasmic signaling tip. Overall, these chemoreceptors have only 32.49% identity in their C-terminal 225 amino acids, but when examining the cytoplasmic tip (equivalent to Tsr amino acids T350-V430), the percent identity increases to 51.28%, and PilJ includes 7 of the 11 conserved residues involved in the association of trimer of dimers (52).

Recent crystallization studies and size exclusion chromatography of the PilJ ligand-binding domain (Q43-R306) showed evidence of trimer formation (53). While trimer formation has been previously reported in another chemoreceptor (54), it is worth noting that PilJ-LBD trimerization occurred independently of the membrane regions or cytoplasmic signaling domain. Should additional studies reveal trimerization of full-length PilJ, it would be possible to determine whether dual adaptors stabilize the trimer oligomerization pattern.

Figure 7B depicts a Chp chemosensory array in *ΔpilI*. Because the ChpC adaptor failed to show interaction with itself or ChpA in this study, we predict that no stable chemosensory complexes form under these circumstances. This correlates with our fluorescent microscopy data and the overwhelming reduction in PilJ-mCherry foci in the *ΔpilI* strain (Fig. 4). Additionally, the absence of chemosensory arrays correlates with the phenotypes reported here and elsewhere: a lack of cAMP production and twitching motility, reduced PilA levels, and loss of surface piliation in *ΔpilI* and *ΔpilIΔchpC* (21) (Fig. 5 and 6).

Figure 7C depicts a Chp chemosensory array in *ΔchpC*. Because PilI and ChpA demonstrate interaction through the BACTH assay and PilJ shows polar localization in this strain (Fig. 3 and 4), we propose that array formation occurs in the absence of ChpC. The only possible hexagonal ring in these arrays contains a 3:3:0 ratio of PilI:ChpA_P5_:ChpC, meaning only two PilJ receptors can be integrated into each ChpA. These arrays, therefore, are expected to be smaller, less efficient and sensitive, and structurally less flexible than WT arrays, correlating with the reduced cAMP and twitching motility seen here and elsewhere (Fig. 5, 21). The small arrays formed in *ΔchpC* appear to be sufficient to modulate wild-type levels of twitching motility in cAMP-replete cells (Fig. 6). Based on the mechanosensory model (41), an actively twitching cell would be a high signal environment, which could explain why the smaller arrays in *ΔchpC(cyaB_R412H_*) can support twitching motility when cAMP is produced independently. This is also consistent with reduced twitching in *ΔchpC* being the result of insufficient cAMP, causing lowered expression of pilus assembly, alignment, and motor genes, resulting in decreased levels of PilA and surface piliation.

Our model of Chp chemosensory array formation aligns with both mechanosensory and chemosensory models of signal transduction (16, 41, 55). In this model, PilJ receives either mechanical or chemical signals and transduces these signals to ChpA either via PilI directly or through ChpC and PilI in series, triggering ChpA autophosphorylation. ChpA-P phosphorylates PilG, resulting in polar localization of PilG at the leading poles of twitching cells (27). PilG-P also modulates CyaB activity to produce cAMP, which binds to Vfr and induces expression of pilus assembly, alignment, and motor genes (21, 56). Our model adds specificity to how the signal is transduced through the signaling array from PilJ to ChpA.

Recently, it has been suggested that ChpC plays a role in chemotaxis and may act to integrate another, as yet unidentified, chemoreceptor into Chp arrays in strains PAK and PA14, transducing signal from that chemoreceptor to ChpA in a complex consisting of chemoreceptor, ChpC, and ChpA (57). In these experiments, loss of ChpC reduced twitching motility in response to BSA/mucin/oligopeptides, without significantly impacting intracellular cAMP, although the authors did not examine twitching motility independent of cAMP. It is important to acknowledge the distinct approaches used to measure cAMP in these manuscripts. The ELISA assay used determined the amount of free cAMP within the cell, while the reporter system determines the relative amount of cAMP bound to Vfr, resulting in transcription. Previous studies in our lab have shown differing results when these two approaches were used on the same strain under the same conditions (unpublished data). Based on the reduced twitching motility, intracellular cAMP, whole-cell PilA, and surface piliation in *ΔchpC* shown here and by others (21), we expect that ChpC and PilJ interact as part of the larger array. However, the BACTH method proved insufficient to draw conclusions regarding PilJ interactions with any baseplate components, and interaction between PilJ and ChpC does not preclude ChpC from interacting with another chemoreceptor. Therefore, we cannot disregard the possibility of a secondary chemoreceptor.

Our experiments using the BACTH system show that ChpC does not interact with the truncated ChpA in any of the possible vector combinations, and that PilI and ChpC interact with each other but not themselves. Therefore, we consider it unlikely that ChpC transduces signal directly to ChpA. If an alternative chemoreceptor interacts with ChpC, it could transduce signal to ChpA via interactions with PilI in extended chemosensory arrays. Only chemoreceptors of the same heptad class can integrate into the arrays containing multiple chemoreceptors (42, 58). PilJ is of the 40H heptad class, and the *P. aeruginosa* genome encodes 20 other 40H heptad class chemoreceptors. However, heptad class is not the only requirement necessary to determine which chemosensory system a chemoreceptor will localize to. It is possible to computationally assign chemoreceptors to specific chemosensory systems based on methylation sites and conserved residues involved in the interaction between the receptor tip and the adaptor protein (23). Based on computational data combining all of these factors for chemoreceptors in *P. aeruginosa*, only PilJ is assigned to the Chp system, with all other 40H heptad class chemoreceptors assigned to either the CheI system (25) or the Wsp system (23). If further experiments show that ChpC integrates a second chemoreceptor into the Chp system, the model presented in this study would require only minor amendments to account for the second chemoreceptor. No amendment to the model of the hexagonal rings of the array baseplate would be required, and ChpC would remain an auxiliary component.

Interaction between distinct CheW paralogs within a signaling array has been previously reported in *Azospirillum brasilense* (43). This soil bacterium uses two of its four chemotaxis systems (Che1 and Che4) to control flagellar-based motility, and paralogous proteins from these two systems can support signal transduction through the same array. Interestingly, bacterial two-hybrid studies revealed that CheW paralogs from Che1 and Che 4 can interact with each other (heterodimerization), and CheW4 shows self-interaction. *A. brasilense* CheW1, however, behaved similarly to PilI and ChpC, in that it failed to demonstrate significantly different self-interaction relative to the negative control (43). The absence of self-interaction and the potential heterodimerization by CheW paralogs in a single chemosensory array baseplate raises broader questions about signal integration and array formation.

In summary, our work has shown that PilI is the primary adaptor in the Chp system, playing an essential role in array formation, and we propose that PilI is the only adaptor to interface directly with the histidine kinase ChpA. While both adaptors are necessary for full functioning of the Chp system, wild-type levels of twitching motility can be achieved through incomplete arrays (*ΔchpC*) when cAMP is provided independent of Chp signal transduction. Together, we used these results to construct a model for Chp array formation consistent with the interactome of each adaptor protein and the localization studies.

## Data Availability

The data that support the findings of this study are available from the corresponding author upon request.

## SUPPLEMENTAL MATERIAL

Supplemental material aem.01286-26-s0001.pdf Tables S1 to S5.

ASM does not own the copyrights to Supplemental Material that may be linked to, or accessed through, an article. The authors have granted ASM a non-exclusive, world-wide license to publish the Supplemental Material files. Please contact the corresponding author directly for reuse.
