# Supplementary material for: Distinct roles of PilI and ChpC in Pseudomonas aeruginosa Chp chemosensory array architecture
Source: Appl Environ Microbiol. 2026 Aug 27;92(9):e01286-26. doi: 10.1128/aem.01286-26 (PMC13599512; doi:10.1128/aem.01286-26)
Supplement: Supplementary file 1 [file aem.01286-26-s0001.pdf]

Table S1: Strains used in this study

Strain	Description	Source
<u>*E. coli* strains</u>		
DH5αMCR	Cloning strain; *fhuA2 Δ(argF-lacZ) U169 phoA glnV44 Φ80 Δ(lacZ)M15 gyrA96 recA1 relA1 endA1 thi-1 hsdR17*	New England BioLabs
DHM1	Reporter strain for Bacterial Adenylate Cyclase Two-Hybrid Assay; *F-, cya-854, recA1, endA1, gyrA96 (Nal r), thi1, hsdR17, spoT1, rfbD1, glnV44(AS)*	Euromedex Bacth System
DHM1 (pKT25-zip pUT18C-zip)	Positive control for Bacterial adenylate cyclase two-hybrid assay	Euromedex Bacth System
S17-1	Donor strain for conjugal mating*; TpR SmR recA thi pro hsdR- M+ RP4 2-Tc::Mu-Km::Tn7 λpir*	D. Saffarini
<u>*P. aeruginosa* strains</u>		
PAO1	wild type (Iglewski Strain)	C. Harwood
PAO1::*lac*P1-*lacZ*	Chromosomal *lacP1* promoter reporter in PAO1 wild type	(1)
Δ*pilI*::*lac*P1-*lacZ*	In frame deletion of *pilI* (PA0410) with 9 amino acids remaining in PAO1::*lac*P1-*lacZ* reporter strain.	This study
Δ*chpC*::*lac*P1-*lacZ*	In frame deletion of *chpC* (PA0415) with 9 amino acids remaining in PAO1::*lac*P1-*lacZ* reporter strain.	(2)
Δ*cyaB*::*lac*P1-*lacZ*	In frame deletion of *cyaB* (PA3217) with 9 amino acids remaining in PAO1::*lac*P1-*lacZ* reporter strain.	(1)
Δ*pilA*::*lac*P1-*lacZ*	In frame deletion of *pilT* (PA0395) in PAO1::*lac*P1-*lacZ* reporter strain.	(1)

<u>Bacterial Two-Hybrid *E. coli* DHM1 Strains</u>

<u>Experimental Strains</u>

pUT18(C) Plasmid	pK(N)T25 Plasmid	Source
pUT18:*chpC*	pKT25:*chpC*	This Study
pUT18:*chpC*	pKT25:*pilI*	This Study
pUT18:*chpC*	pKT25:*chpA* $_{AA1668\text{-}2349}$	This Study
pUT18:*chpC*	pKNT25:*chpC*	(2)
pUT18:*chpC*	pKNT25:*pilI*	(2)
pUT18:*chpC*	pKNT25:*chpA* $_{AA1668\text{-}2349}$	This Study
pUT18:*pilI*	pKT25:*pilI*	This Study
pUT18:*pilI*	pKT25:*chpC*	This Study
pUT18:*pilI*	pKT25:*chpA* $_{AA1668\text{-}2349}$	This Study
pUT18:*pilI*	pKNT25:*pilI*	(2)
pUT18:*pilI*	pKNT25:*chpC*	(2)
pUT18:*pilI*	pKNT25:*chpA* $_{AA1668\text{-}2349}$	This Study
pUT18:*chpA* $_{AA1668\text{-}2349}$	pKT25:*chpA* $_{AA1668\text{-}2349}$	This Study
pUT18:*chpA* $_{AA1668\text{-}2349}$	pKT25:*pilI*	This Study
pUT18:*chpA* $_{AA1668\text{-}2349}$	pKT25:*chpC*	This Study
pUT18:*chpA* $_{AA1668\text{-}2349}$	pKNT25:*chpA* $_{AA1668\text{-}2349}$	This Study
pUT18:*chpA* $_{AA1668\text{-}2349}$	pKNT25:*pilI*	This Study
pUT18:*chpA* $_{AA1668\text{-}2349}$	pKNT25:*chpC*	This Study

pUT18C:*chpC*	pKT25:*chpC*	This Study
pUT18C:*chpC*	pKT25:*pilI*	This Study
pUT18C:*chpC*	pKT25:*chpA* $_{AA1668\text{-}2349}$	This Study
pUT18C:*chpC*	pKNT25:*chpC*	This Study
pUT18C:*chpC*	pKNT25:*pilI*	This Study
pUT18C:*chpC*	pKNT25:*chpA* $_{AA1668\text{-}2349}$	This Study
pUT18C:*pilI*	pKT25:*pilI*	This Study
pUT18C:*pilI*	pKT25:*chpC*	This Study
pUT18C:*pilI*	pKT25:*chpA* $_{AA1668\text{-}2349}$	This Study
pUT18C:*pilI*	pKNT25:*pilI*	This Study
pUT18C:*pilI*	pKNT25:*chpC*	This Study
pUT18C:*pilI*	pKNT25:*chpA* $_{AA1668\text{-}2349}$	This Study
pUT18C:*chpA* $_{AA1668\text{-}2349}$	pKT25:*chpA* $_{AA1668\text{-}2349}$	This Study
pUT18C:*chpA* $_{AA1668\text{-}2349}$	pKT25:*pilI*	This Study
pUT18C:*chpA* $_{AA1668\text{-}2349}$	pKT25:*chpC*	This Study
pUT18C:*chpA* $_{AA1668\text{-}2349}$	pKNT25:*chpA* $_{AA1668\text{-}2349}$	This Study
pUT18C:*chpA* $_{AA1668\text{-}2349}$	pKNT25:*pilI*	This Study
pUT18C:*chpA* $_{AA1668\text{-}2349}$	pKNT25:*chpC*	This Study
<u>Control Strains</u>		
pUT18:*chpC*	pKT25	This Study
pUT18:*chpC*	pKNT25	This Study
pUT18:*pilI*	pKT25	This Study
pUT18:*pilI*	pKNT25	This Study
pUT18:*chpA* $_{AA1668\text{-}2349}$	pKT25	This Study
pUT18:*chpA* $_{AA1668\text{-}2349}$	pKNT25	This Study
pUT18C:*chpC*	pKT25	(2)
pUT18C:*chpC*	pKNT25	This Study
pUT18C:*pilI*	pKT25	(2)
pUT18C:*pilI*	pKNT25	This Study
pUT18C:*chpA* $_{AA1668\text{-}2349}$	pKT25	This Study
pUT18C:*chpA* $_{AA1668\text{-}2349}$	pKNT25	This Study
pUT18	pKT25:*pilI*	This Study
pUT18	pKT25:*chpC*	This Study
pUT18	pKT25:*chpA* $_{AA1668\text{-}2349}$	This Study
pUT18	pKNT25:*pilI*	(2)
pUT18	pKNT25:*chpC*	(2)
pUT18	pKNT25:*chpA* $_{AA1668\text{-}2349}$	This Study
pUT18C	pKT25:*pilI*	This Study
pUT18C	pKT25:*chpC*	This Study
pUT18C	pKT25:*chpA* $_{AA1668\text{-}2349}$	This Study
pUT18C	pKNT25:*pilI*	This Study
pUT18C	pKNT25:*chpC*	This Study
pUT18C	pKNT25:*chpA* $_{AA1668\text{-}2349}$	This Study
pUT18	pKT25	This Study
pUT18	pKNT25	(2)
pUT18C	pKT25	This Study
pUT18C	pKNT25	This Study
pUT18C-zip	pKT25-zip	(2)

Table S2: Plasmids used in this study

Plasmid	Description	Source or reference
pEX18Tc	Gene replacement suicide vector, Tet^R	(3)
pEX18Tc:Δ*chpC*	pEX18Tc based plasmid for deletion of *chpC*	(2)
pEX18Tc:Δ*pilI*	pEX18Tc based plasmid for deletion of *pilI*	This Study
pJN105	Expression Vector; Gm^R	(4)
pJN105:*cyaB*$_{R412H}$	Constitutively active adenyl cyclase *cyaB* cloned into pJN105 at EcoRI and XbaI sites	(5)
pSB109	Arabinose inducible expression vector, Gm^R	(6)
pSB109:*chpC*	*chpC* cloned into pSB109 at EcoRI and SmaI sites	(2)
pSB109:*pilI*	*pilI* cloned into pSB109 at EcoRI and HindIII sites	(2)
pUT18	Bacterial adenylate cyclase two hybrid vector, Amp^R	Euromedex Bacth System
pUT18:*chpA*$_{AA1668\text{-}2349}$	*chpA* amino acids 1668-2349 fused in frame at the N-terminus of T18 fragment of the pUT18 plasmid at the XbaI and XmaI sites.	This Study
pUT18:*chpC*	*chpC* fused in frame at the N-terminus of T18 fragment of the pUT18 plasmid at the PstI and EcoRI sites	(2)
pUT18:*pilI*	*pilI* fused in frame at the N-terminus of the T18 fragment of the pUT18 plasmid at the PstI and EcoRI sites	(2)
pUT18:*pilJ*	*pilJ* fused in frame at the N-terminus of the T18 fragment of the pUT18 plasmid at the BamHI and EcoRI sites	(2)
pUT18C	Bacterial adenylate cyclase two hybrid vector, Amp^R	Euromedex Bacth System
pUT18C:*chpA*$_{AA1668\text{-}2349}$	*chpA* amino acids 1668-2349 fused in frame at the C-terminus of T18 fragment of the pUT18C plasmid at the XbaI and XmaI sites.	This Study
pUT18C:*chpC*	*chpC* fused in frame at the C-terminus of T18 fragment of the pUT18C plasmid at the PstI and BamHI sites	This Study
pUT18C:*pilI*	*pilI* fused in frame at the C-terminus of T18 fragment of the pUT18C plasmid at the PstI and BamHI sites	This Study

pUT18C:*pilJ*	*pilJ* fused in frame at the C-terminus of the T18 fragment of the pUT18C plasmid at the BamHI and EcoRI sites	This Study
pKNT25	Bacterial adenylate cyclase two hybrid vector, Kan^R	Euromedex Bacth System
pKNT25:$chpA_{AA1668-2349}$	*chpA* amino acids 1668-2349 fused in frame at the N-terminus of T25 fragment of the pKNT25 plasmid at the XbaI and XmaI sites	This Study
pKNT25:*chpC*	*chpC* fused in frame at the N-terminus of T25 fragment of the pKNT25 plasmid at the PstI and EcoRI sites	(2)
pKNT25:*pilI*	*pilI* fused in frame at the N-terminus of T25 fragment of the pKNT25 plasmid at the PstI and EcoRI sites	(2)
pKNT25:*pilJ*	*pilJ* fused in frame at the N-terminus of T25 fragment of the pKNT25 plasmid at the BamHI and EcoRI sites	(2)
pKT25	Bacterial adenylate cyclase two hybrid vector, Kan^R	Euromedex Bacth System
pKT25:$chpA_{AA1668-2349}$	*chpA* amino acids 1668-2349 fused in frame at the C-terminus of T25 fragment of the pKT25 plasmid at the XbaI and XmaI sites	This Study
pKT25:*chpC*	*chpC* fused in frame at the C-terminus of T25 fragment of the pKT25 plasmid at PstI and BamHI sites	This Study
pKT25:*pilI*	*pilI* fused in frame at the C-terminus of T25 fragment of the pKT25 plasmid at PstI and BamHI sites	This Study
pKT25:*pilJ*	*pilJ* fused in frame at the C-terminus of T25 fragment of pKT25 at BamHI and EcoRI sites	This Study

Table S3: Primers used in this study

	Primer	Sequence (5'-3')[a]
In-frame deletions	*ΔchpC*_UPFor	TGCCGGAATTCGCAGCGCCACGTGTTGCA
	*ΔchpC*_UPRev	GCTCAGATCAGGCCGGCGTCGGCCTGGTTCATGTTTCG
	*ΔchpC*_DNFor	CGAAACATGAACCAGGCCGACGCCGGCCTGATCTGAGC
	*ΔchpC*_DNRev	ACAAAGCTTGGAAGCCGCCGGTCAAACCG
	*ΔcyaB*_UPFor	AGGTACCCTTCCGCGATGATCCGCTGG
	*ΔcyaB*_UPRev	GCGCTGGAGAGGATCCCTG
	*ΔcyaB*_DNFor	GCGACCTCTCCTAGGGACGTTCGTCGAACGCCGCCGGCA
	*ΔcyaB*_DNRev	ACTGCAGGTCGTCACCAGCCTGCTGG
	*ΔpilA*_UPFor	CGCGAATTCCGTCCTGCGGTTTGCG
	*ΔpilA*_UPRev	CAAGCCACCTTCGATCACCGCATCTCTCCGTTGATTATG
	*ΔpilA*_DNFor	CATAATCAACGGAGAGATTCGGTGATCGAAGGTGGCTTG
	*ΔpilA*_DNRev	GTCCTGCAGCGGCGGCGACCTTACC
	*ΔpilI*_UPFor	CAGGAATTCGGCTCGCGATTGGGTCGC
	*ΔpilI*_UPRev	TACGGCGACGTCGAGGAAGTCCGACAGGCGGGGCCTG
	*ΔpilI*_DNFor	CAGGCCCCGCATGTCGGACTTCCTCGACGTCGCCGTA
	*ΔpilI*_DNRev	CAGAAGCTTGATGTTGTTCGCCGCTTC
	*ΔpilT*_UPFor	TGGATCCTTGCCGGCGCCGATGAACG
	*ΔpilT*_UPRev	GCGGCGGATCGGCGCCGCCAGGAGGGACTCCCCAATTACAAGCAAG
	*ΔpilT*_DNFor	TGCTTGTAATTGGGGAGTCCCTCCTGGCGCCGATCCGCCGC
	*ΔpilT*_DNRev	GAAGCTTGACAGGTCCATCCACACCTG
	*ΔcpdA*_UPFor	GCTAGGAATTCGGTGCTCATCGAGCAATTCCG
	*ΔcpdA*_UPRev	GCATGTCAGTATCCGGCGGTTGACAAGGGGCCGTCTCCTG
	*ΔcpdA*_DNFor	CAGGAGACGGCCCCTTGTCAACCGCCGGATACTGACATGC
	*ΔcpdA*_DNRev	GCTAGAAGCTTGGTAAGGATCAGCTCGACGCC
Expression	*chpC*-For	CATGCGAATTCATGAACCAGGCCGTGATC
	chpC-Rev	GTATCCCGGGTCAGATCAGGCCGGCGTC
	pilI-For	GTACAGAATTCATGTCGGACGTTCAGACC
	pilI-Rev	CGCGCAAGCTTTTATACGGCGACGTCGAG
	cyaB-For	AGGAATTCATGAAGCCTACCCTCCCCGAC
	cyaB-Rev	ATTCTAGATTAGAGGATGACCTTGTCGCG
	cyaB$_{R412H}$-For	CAGGTGGTGGACTCGCACCGCGACCTCGGCGCC
	cyaB$_{R412H}$-Rev	GGCGCCGAGGTCGCGGTGCGAGTCCACCACCTG
Bacterial Two-Hybrid	*pilI*_FR-pKT25	GTACTGCAGGGATGTCGGACGTTCAGACCCCCTTC
	*pilI*_FR-KNT-UT-C	GTTCTGCAGGATGTCGGACGTTCAGACCCCCTTC
	*pilI*_RV-KT-UTC	CGGGGGATCCTCTTATACGGCGACGTCGAGGAA
	*pilI*_RV-KNT-UT	CAGGGATCCTCTACGGCGACGTCGAGGAAGCC
	*chpC*_FR-pKT25	GATGCTGCAGGGATGAACCAGGCCGTGATCGAGC

	*chpC*_FR-KNT-UT-C	GAAGCCTGCAGGATGAACCAGGCCGTGATCGAGC
	*chpC*_RV-KT-UTC	CAAGGGATCCTCTCAGATCAGGCCGGCGTCGGC
	*chpC*_RV-KNT-UT	CAGGGATCCTCGATCAGGCCGGCGTCGGCGAG
	*pilJ*_FR	GTCGGATCCCATGAAGAAAATCAACGCAG
	*pilJ*_RV-pKT25	CATTGAATTCTTCAGGCCTGCTCCACGCC
	*pilJ*_RV-KNT-UT/C	GTTAGAATTCGAGGCCTGCTCCACGCCCTC
	*chpA*_FR	GTAACTCTAGACGACGAGAGCGAGATCCT
	*chpA*_RV	GAATACCCGGGGCCGCTGCTGCTCGCTTTC

[a]Restriction enzyme sites are underlined

Table S4: Distribution of Tfp-class chemosensory system CheW proteins among Gammaproteobacteria.

	Clade	Taxonomic Orders (NCBI)	Species	Tfp-class system	# of Tfp CheWs	CheW1 (PilI) Accession	CheW2 (ChpC) Accession	CheW3 (PilI) Accession	CheW4 (ChpC) Accession	Notes
Gammaproteobacteria	Entero	Enterobacteriales	Yersinia pestis KIM	No						
	Entero	Enterobacteriales	Yersinia pestis CO92	No						
	Entero	Enterobacteriales	Serratia proteamaculans 568	No						
	Entero	Enterobacteriales	Photorhabdus luminescens BA1	No						
	Entero	Enterobacteriales	Pectobacterium atrosepticum SCRI1043	No						
	Entero	Enterobacteriales	Sodalis glossindius	No						
	Entero	Enterobacteriales	Salmonella enterica Typhi str. CT18	No						
	Entero	Enterobacteriales	Salmonella typhimurium LT2	No						
	Entero	Enterobacteriales	Escherichia coli K12	No						
	Entero	Enterobacteriales	Enterobacter sp. 638	No						
	Entero	Enterobacteriales	Buchnera aphidicola str. APS	No						
	Entero	Enterobacteriales	Buchnera aphidicola str. Sg	No						
	Entero	Enterobacteriales	B. aph. Cc	No						
	Entero	Enterobacteriales	Buchnera aphidicola str. Bp	No						
	VAAP	Pasteurellales	Pasteurella multocida str. Pm70	No						
	VAAP	Pasteurellales	Haemophilus somnus 2336	No						
	VAAP	Pasteurellales	Haemophilus influenzae Rd KW20	No						
	VAAP	Pasteurellales	Actinobacillus succinogenes 130Z	No						
	VAAP	Pasteurellales	Mannheimia succiniciproducens MBEL55E	No						
	VAAP	Pasteurellales	Actinobacillus pleuropneumoniae L20	No						
	VAAP	Pasteurellales	Haemophilus ducreyi 35000HP	No						
	VAAP	Vibrionales	Vibrio alginolyticus 12G01	No						
	VAAP	Vibrionales	Vibrio parahaemolyticus RIMD 2210633	No						
	VAAP	Vibrionales	Vibrio vulnificus YJ016	No						
	VAAP	Vibrionales	Vibrio shilonii AK1	No						
	VAAP	Vibrionales	Vibrionales bacterium SWAT-3	No						
	VAAP	Vibrionales	Vibrio cholerae O1 str. N16961	No						
	VAAP	Vibrionales	Vibrio fischeri ES114	No						
	VAAP	Vibrionales	Photobacterium angustum S14	No						
	VAAP	Vibrionales	Photobacterium profundum SS9	No						
	VAAP	Diverse Singleton Orders	Aeromonas hydrophila ATCC 7966	No						
	VAAP	Alteromonadales	Psychromonas ingrahamii 37	No						
	VAAP	Alteromonadales	Psychromonas sp. CNPT3	No						
	VAAP	Alteromonadales	Moritella sp. PE36	No						
	VAAP	Alteromonadales	Shewanella putrefaciens CN-32	No						
	VAAP	Alteromonadales	Shewanella baltica OS155	No						
	VAAP	Alteromonadales	Shewanella oneidensis MR-1	No						
	VAAP	Alteromonadales	Shewanella denitrificans OS217	No						
	VAAP	Alteromonadales	Shewanella frigidimarina NCIMB 400	No						
	VAAP	Alteromonadales	Shewanella woodyi ATCC 51908	No						
	VAAP	Alteromonadales	Shewanella pealeana ATCC 700345	No						
	VAAP	Alteromonadales	Shewanella loihica PV-4	No						
	VAAP	Alteromonadales	Shewanella amazonensis SB2B	No						
	VAAP	Alteromonadales	Pseudoalteromonas haloplanktis TAC125	No						
	VAAP	Alteromonadales	Alteromonadales bacterium TW-7	No						
	VAAP	Alteromonadales	Pseudoalteromonas tunicata D2	No						
	VAAP	Alteromonadales	Colwellia psychrerythraea 34H	No						
	VAAP	Alteromonadales	Idiomarina loihiensis L2TR	No						
	VAAP	Alteromonadales	Idiomarina baltica OS145	No						
	VAAP	Alteromonadales	Alteromonas macleodii ATCC 27126	No						
	VAAP	Alteromonadales	Pseudoalteromonas atlantica T6c	No						
	PO	Pseudomonadales	Pseudomonas fluorescens F113	Yes	2	WP_014340665.1	WP_014340662.1			
	PO	Pseudomonadales	Pseudomonas fluorescens PfO-1	Yes	2	WP_011336364.1	WP_011336361.1			
	PO	Pseudomonadales	Pseudomonas syringae DC3000	Yes	2	WP_011105281.1	WP_005769407.1			
	PO	Pseudomonadales	Pseudomonas putida KT2440	Yes	2	WP_010955559.1	WP_003249336.1			
	PO	Pseudomonadales	Pseudomonas entomophila L48	Yes	2	WP_011536046.1	WP_011536043.1			
	PO	Pseudomonadales	Pseudomonas stutzeri A1501	Yes	2	WP_013984316.1	WP_011914952.1			
	PO	Pseudomonadales	Pseudomonas mendocina ymp	Yes	2	WP_011920657.1	WP_011920662.1			
	PO	Pseudomonadales	Pseudomonas aeruginosa PAO1	Yes	2	NP_249101.1	NP_249106.1			
	PO	Oceanospirillales	Chromohalobacter salexigens DSM 3043	No						
	PO	Alteromonadales	Marinobacter hydrocarbonoclasticus VT8	Yes	2	WP_011787172.1	WP_011787177.1			
	PO	Alteromonadales	Marinobacter sp. ELB17	Yes	2	WP_007348234.1	WP_007348229.1			
	PO	Oceanospirillales	Hahella chejuensis KCTC 2396	Yes, two	4 (two sets)	WP_011394526.1	WP_011394521.1	WP_011399538.1	WP_011399543.1	Two Tfp operons
	PO	Oceanospirillales	Marinomonas sp. MWYL1	No						
	PO	Oceanospirillales	Marinomonas sp. MED121	No						
	PO	Oceanospirillales	Neptuniibacter caesariensis	Yes	1	WP_007021546.1				
	PO	Oceanospirillales	Bermanella marisrubri	Yes	2	WP_007016689.1	WP_007016694.1			
	PO	Unassigned	Reinekea blandensis MED297	Yes	2	WP_008047944.1	WP_008047935.1			
	PO	Unassigned	Congregibacter litoralis KT71	No						
	PO	Unassigned	marine gamma proteobacterium HTCC2080	No						
	PO	Unassigned	marine gamma proteobacterium HTCC2143	Yes	2	WP_007224229.1	WP_007224233.1			
	PO	Unassigned	marine gamma proteobacterium HTCC2207	No						
	PO	Alteromonadales	Saccharophagus degradans 2-40	Yes, two	3 (two sets)	WP_011470102.1	WP_011470097.1	WP_011467006.1		Two Tfp operons. Second operon has some weird variants of the histidine kinase and MCP
	PO	Pseudomonadales	Acinetobacter sp. ADP1	Yes	1	WP_004922368.1				
	PO	Pseudomonadales	Acinetobacter baumannii ATCC 19606	Yes	1	WP_000729758.1				
	PO	Pseudomonadales	Psychrobacter sp. PRwf-1	Yes	1	WP_011961370.1				
	PO	Pseudomonadales	Psychrobacter cryohalentis K5	Yes	1	WP_011514414.1				
	PO	Oceanospirillales	Alcanivorax borkumensis SK2	Yes	2	WP_011587405.1	WP_011587400.1			
	Basal	Unassigned	Ruthia magnifica str. Cm	No						
	Basal	Unassigned	Vesicomyosocius okutanii HA	No						
	Basal	Thiotrichales	Hydrogenovibrio crunogena XCL-2	No						
	Basal	Diverse Singleton Orders	Dichelobacter nodosus VCS1703A	Yes	1	WP_012031399.1				
	Basal	Thiotrichales	Francisella tularensis SCHU S4	No						
	Basal	Legionellales	Coxiella burnetii RSA 493	No						
	Basal	Legionellales	Rickettsiella grylli	No						
	Basal	Legionellales	Legionella pneumophila Philadelphia 1	No						
	Basal	Thiotrichales	Beggiatoa alba B18LD	Yes, two	4 (two sets)	WP_002691297.1	WP_002691285.1	WP_002691213.1	WP_002691204.1	Two Tfp operons
	Basal	Chromatiales	Nitrosococcus oceani ATCC 19707	Yes	2	WP_002812178.1	WP_002814031.1			
	Basal	Diverse Singleton Orders	Methylococcus capsulatus str. Bath	No						
	Basal	Unassigned	Endoriftia persephonae	Yes	2	WP_240991742.1	WP_005965736.1			
	Basal	Chromatiales	Alkalilimnicola ehrlichei MLHE-1	Yes	2	WP_049753504.1	WP_011628111.1			
	Basal	Chromatiales	Halorhodospira halophila SL1	No						
	Basal	Chromatiales	Nitrococcus mobilis Nb-231	Yes	2	WP_005003427.1	WP_005003432.1			
	Basal	Xanthomonadales	Xanthomonas campestris ATCC 33913	Yes	2	WP_011038046.1	WP_011038042.1			
	Basal	Xanthomonadales	Xanthomonas citri pv. Viginicola	Yes	2	WP_003482487.1	WP_003482475.1			
	Basal	Xanthomonadales	Xylella fastidiosa 9a5c	Yes	2	WP_004085458.1	WP_004085454.1			
	Basal	Xanthomonadales	Stenotrophomonas maltophilia K279a	Yes	2	WP_005410716.1	WP_005410712.1			
Non-Gamma	Non-Gamma	Outgroup	Ralstonia solanacearum GMI1000	Yes	1	WP_011000625.1				Tfp systems with single adaptors seem to be common in Burkholderia relatives
	Non-Gamma	Outgroup	Chromobacterium violaceum ATCC 12472	No						
	Non-Gamma	Diverse Singleton Orders	Acidithiobacillus ferrooxidans ATCC 23270	No						
	Non-Gamma	Unassigned	Mariprofundus ferrooxydans PV-1	No						
	Non-Gamma	Outgroup	Rhodospirillum rubrum ATCC 11170	No						
	Non-Gamma	Outgroup	Sinorhizobium meliloti 1021	No						

Table S5: Table S4: Distribution of Tfp-class chemosensory system CheW proteins among Betaproteobacteria.

Order	Species	Tfp-class system	# of CheWs	CheW1 Accession	CheW2 Accession	Gene Neighborhood								Notes
Neisseriales	Vitresoscilla filiformis	Yes	1	WP_089417722.1		<CheA	<MCP	<CheW	<RR2	<RR1				
Burkholderiales	Comamonas testosteroni TK 102	Yes	1	WP_003051512.1		<CheA	<MCP	<CheW	<RR2	<RR1				
Burkholderiales	Burkholderia cepacia ATCC 25416	No												
Burkholderiales	Ralstonia solanacearum GMI 1000	Yes	1	WP_011000625.1		RR1>	RR2>	CheW>	MCP>	CheA>				
Burkholderiales	Ralstonia pickettii 12J	Yes	1	WP_004629641.1		RR1>	RR2>	CheW>	MCP>	CheA>				
Neisseriales	Neisseria gonorrhoeae FA 1090	No												
Neisseriales	Neisseria lactamica 020-06	No												
Burkholderiales	Alcaligenes faecalis	No												
Burkholderiales	Bordetella bronchiseptica 253	No												
Burkholderiales	Bordetella pertussis Tohama I	No												
Neisseriales	Chromobacterium violaceum ATCC 12472	No												
Burkholderiales	Oxalobacter formigenes	No												
Nitrosomonadales	Nitrosomonas europaea ATCC 19718	Yes	0			<CheA	<MCP							
Burkholderiales	Thiomonas intermedia	Yes	1	WP_079418542.1		<CheA	<MCP	<CheW	<RR2	<RR1				
Burkholderiales	Herbaspirillum seropedicae	Yes	1	WP_013235781.1		<CheA	<MCP	<CheW						
Nitrosomonadales	Nitrosospira multiformis	No												
Burkholderiales	Burkholderia mallei ATCC 23344	No												
Burkholderiales	Rubrivivax gelatinosus IL 144	Yes	1	WP_014430394.1		<GGDEF	<CheA	<MCP	<CheW	<RR2	<RR1			GGDEF = "GGDEF domain-containing protein" unclear if part of operon
Burkholderiales	Tayorelle equigenitalis ATCC 35865	No												
Burkholderiales	Leptothrix cholodnii SP-6	Yes	1	WP_012348968.1		<GGDEF	<CheA	<MCP	<CheW	<RR2	<RR1			
Burkholderiales	Variovorax paradoxus EPS	Yes	1	WP_013543586.1		<CheA	<MCP	<CheW	<RR2	<RR1				
Nitrosomonadales	Thiobacillus denitrificans ATCC 25259	Yes	1	WP_011313062.1		<CheA	<MCP	<CheW	<RR2	<RR1				
Burkholderiales	Paraburkholderia xenovorans LB400	No												
Burkholderiales	Janthinobacterium agaricidamnosum NBRC 102515 = DSM 9628	Yes	1	WP_051780249.1		RR1>	RR2>	CheW>	MCP>	CheA>				
Nitrosomonadales	Sideroxydans lithotrophicus ES-1	Yes	1	WP_013031059.1		<CheA	<MCP	<CheW	<RR2	<RR1				
Burkholderiales	Cupravidus basilensis	Yes	1	WP_043344419.1		RR1>	RR2>	CheW>	MCP>	CheA>				
Burkholderiales	Castellaniella defragrans 65Phen	No												
Burkholderiales	Roseateles depolymerans	Yes	1	WP_058936516.1		<GGDEF	<CheA	<MCP	<CheW	<RR2	<RR1			
Burkholderiales	Delftia acidovorans SPH-1	Yes	1	WP_012203503.1		RR1>	RR2>	CheW>	MCP>	CheA>				
Burkholderiales	Acidovorax avenae subsp. Avenae ATCC 19860	Yes	1	WP_013593320.1		RR1>	RR2>	CheW>	MCP>	CheA>				
Burkholderiales	Rhodoferax antarcticus	Yes	1	WP_075584814.1		<CheA	<MCP	<CheW	<RR2	<RR1				
Burkholderiales	Achromobacter xylosoxidans	No												
Burkholderiales	Herbaspirillum frisingense	Yes	1	WP_079219324.1		RR1>	RR2>	CheW>	MCP>	CheA>				
Burkholderiales	Pandoraea pnomenusa	No												
Burkholderiales	Ramlibacter tataouinensis TTB310	Yes, two	1	WP_013902759.1		<CheA	<MCP	<CheW	<RR2	<RR1				
			2	WP_081466252.1	WP_081466253.1	RR1>	RR2>	RR3>	CheW1>	MCP>	CheA>	???>	CheW2>	??? = "hypothetical protein" no annotated homologues appeared with protein BLAST
Rhodocyclales	Thauera chlorobenzoica	No												
Burkholderiales	Methylibium petroleiphilum PM1	Yes	1	WP_011830764.1		<CheA	<MCP	<CheW	<RR2	<RR1				
Rhodocyclales	Dechlorosoma suillum PS	Yes	1	WP_014235769.1		<CheA	<MCP	<CheW	<RR2	<RR1				
Neisseriales	Laribacter hongkongensis HLHK9	No												
Burkholderiales	Alicycliphilus denitrificans K601	Yes	1	WP_013520428.1		RR1>	RR2>	CheW>	MCP>	CheA>				
Burkholderiales	Herminiimonas arsenicoxydans	Yes	1	WP_041322637.1		<CheA	<MCP	<CheW	<RR2	<RR1				
Burkholderiales	Polaromonas naphthalenivorans CJ2	Yes	1	WP_011802720.1		<CheA	<MCP	<CheW	<RR2	<RR1				
Rhodocyclales	Dechloromonas aromatica RCB	Yes	1	WP_011289620.1		<CheA	<MCP	<CheW	<RR2	<RR1				
Nitrosomonadales	Methylovorus glucosetrophus SIP3-4	Yes	1	WP_015830396.1		<CheA	<MCP	<CheW	<RR2	<RR1				
Burkholderiales	Collimonas pratensis	Yes	1	WP_061937386.1		RR1>	RR2>	CheW>	MCP>	CheA>				
Burkholderiales	Advenella mimigardefordensis DPN7	No												
Nitrosomonadales	Methylotenera mobilis JLW8	Yes	1	WP_015832233.1		<CheA	<MCP	<CheW	<RR2	<RR1				
Burkholderiales	Verminephrobacter eiseniae EF01-2	Yes	1	WP_011810747.1		<CheA	<MCP	<CheW	<RR2	<RR1				
Nitrosomonadales	Gallionella capsiferriformans ES-2	No												
Rhodocyclales	Azoarcus olearius	Yes	1	WP_065341288.1		RR1>	RR2>	CheW>	MCP>	CheA>				
Rhodocyclales	Aromatoleum aromaticum EbN1	Yes	1	WP_011236796.1		RR1>	RR2>	CheW>	MCP>	CheA>				
Burkholderiales	Polynucleobacter necessarius STIR1	No												
Burkholderiales	Paenalcaligenes hominis	No												
Nitrosomonadales	Sulfuricella denitrificans skB26	Yes	1	WP_009207316.1		<CheA	<MCP	<CheW	<RR2	<RR1				
Nitrosomonadales	Sulfuritalea hydrogenivorans sk43H	Yes	1	WP_041100972.1		RR1>	RR2>	CheW>	MCP>	CheA>	VOC>			VOC = "VOC family protein" unclear if part of operon
Burkholderiales	Rhizobacter gummiphilus	Yes	1	WP_085753028.1		<GGDEF	<CheA	<MCP	<CheW	<RR2	<RR1			
Burkholderiales	Massilia putida	Yes	1	WP_075793279.1		<CheA	<MCP	<CheW	<RR2	<RR1				
Neisseriales	Snodgrassella alvi wkB2	No												
Burkholderiales	Basilea psittacipulmonis DSM 24701	No												
Burkholderiales	Diaphorobacter polyhydroxybutyrativorans	Yes	1	WP_011806673.1		RR1>	RR2>	CheW>	MCP>	CheA>				

References

1. Jansari VH. 2017. The role of PilJ and its structural domains in the localization and function of the Chp chemosensory system in. THESIS.DOCTORAL. University of Wisconsin Milwaukee.

2. Sharma S. 2018. Role of the two adaptor proteins in the Chp chemosensory system of Pseudomonas aeruginosa. University of Wisconsin Milwaukee.

3. Hoang T, Karkhoff-Schweizer R, Kutchma A, Schweizer H. 1998. A broad-host-range Flp-FRT recombination system for site-specific excision of chromosomally-located DNA sequences: application for isolation of unmarked Pseudomonas aeruginosa mutants. Gene 212:77–86.

4. Newman JR, Fuqua C. 1999. Broad-host-range expression vectors that carry the L-arabinose-inducible Escherichia coli araBAD promoter and the araC regulator. Gene 227:197–203.

5. Jansari VH, Potharla VY, Riddell GT, Bardy SL. 2016. Twitching motility and cAMP levels: signal transduction through a single methyl-accepting chemotaxis protein. FEMS Microbiology Letters 363.

6. Ketelboeter LM, Bardy SL. 2017. Characterization of 2-(2-nitro-4-trifluoromethylbenzoyl)-1,3-cyclohexanedione resistance in pyomelanogenic Pseudomonas aeruginosa DKN343. Plos One 12:e0178084.
